# Why Bigger Is Not Yet Better: The Problems with Huge Datasets

**DOI:** 10.1371/journal.pmed.0020055

**Published:** 2005-02-22

**Authors:** The PLoS Medicine Editors

## Abstract

Traditional two dimensional journal publishing may no longer be enough, and may be hindering, the interpretation of the complex datasets and analyses generated by microarray experiments in medicine

“Publishing results in traditional paper based way in a journal hides too much information.” This is the verdict of Markus Ruschhaupt and colleagues who, in a paper in *Statistical Applications in Genetics and Molecular Biology* (3: article 37), discuss a paradigm for the presentation of complex data—in this case, from microarray analyses. The title of the article, “A Compendium to Ensure Reproducibility in High-Dimensional Classification Tasks,” may not lend itself easily to a clinical audience, but the underlying message to clinicians could not be more important: that, currently, studies involving large datasets, especially ones that have a clinical outcome, are so poorly reported (or possibly so poorly done) that many are not reproducible. This problem was also the topic of a recent meeting in Heidelberg, “Best Practice in Microarray Studies” (http://www.biometrie.uni-heidelberg.de/workshops/bestpractice/index.htm).

As microarrays have become mainstream research tools in biology and medicine, the large datasets and complex analyses from these studies have presented challenges: for authors in analyzing the data, for reviewers and editors in deciding on the suitability of papers for publication, for journals in determining how much data needs to be presented within the paper itself, for other researchers in reproducing the data, and, finally, for readers in deciding how to assess the data presented. The results from several high-profile papers have already proved difficult to reproduce, even by those with sufficient time and computing expertise.

Where do such analyses leave the new science of molecular pathology? Ruschhaupt and colleagues comment that “the literature on the induction of prognostic profiles from microarray studies is a methodological wasteland.” Much the same could be said of other applications of molecular biology to clinical samples. A systematic review of molecular and biological tumor markers in neuroblastoma (Clin Cancer Res 10: 4–12) found that its conclusions were limited by “small sample sizes, poor statistical reporting, large heterogeneity across studies…and publication bias.” John Ioannidis and colleagues (Lancet 362: 1439–1444) did a similar analysis of 30 microarray studies with major clinical outcomes in cancer. They showed that the studies were small—median sample size was 25 patients, and validation was incomplete in most studies. They recommended that molecular prognostic studies be classified as phase 1 (early exploratory probing associations), phase 2 (exploratory with extensive analyses), or phase 3 (large confirmatory studies with pre-stated hypotheses and precise quantification of the magnitude of the effect), and that only studies that had undergone phase 3 testing should be considered robust enough for use in clinical practice. Most current studies should be considered as phase 1 or, at best, phase 2.

So, despite considerable hype, the published studies are far from the level of evidence that would be accepted for virtually any other medical test. In a review in 2003 (Hematology [Am Soc Hematol Educ Program] 2003: 279–293), Rita Braziel and colleagues concluded, “rapid identification and neutralization of spurious results is essential to prevent them from becoming accepted facts.”

But these problems are not new in medical research. In 1994 (BMJ 308: 283–284), Doug Altman, who was instrumental in developing the CONSORT guidelines for reporting of clinical trials, said that “huge sums of money are spent annually on research that is seriously flawed through the use of inappropriate designs, unrepresentative samples, small samples, incorrect methods of analysis, and faulty interpretation,” and “that quality control needs to be built in from the start rather than the failures being discarded.”

So how can we ensure that the wealth of data pouring out of microarray and other molecular diagnostic studies is turned into meaningful knowledge? The Microarray Gene Expression Data Society has proposed a set of guidelines (MIAME) for the reporting of microarray data, and that all such data should be deposited in public databases. But as Ruschhaupt and others have shown, disclosure of results and data is not enough, since there is little consensus on the appropriate statistical analyses and many are developed on a case by case basis, which may not be reproducible, even by the authors. Some researchers advocate the use of standard statistical packages, which allows the reader to repeat an entire analysis quickly and, hence, assess the robustness of the results. Some authors have produced a transcript of their statistical analyses as a supplement to their articles (e.g., Nucleic Acids Res 32: e50). At the very least authors should have a protocol with a prespecified plan for patient selection and statistical analysis—accepted practice for clinical trials, but not yet for other medical research. An ultimate aim for reporting would be the type of compendium discussed by Ruschhaupt and colleagues—“an interactive document that bundles primary data, statistical processing methods, figures, and derived data together with the textual documentation and conclusions.” One such compendium is illustrated in a paper by Robert Gentleman (Stat Appl Genet Mol Biol 4: article 2). *PLoS Medicine* is keen to work with authors towards making such reporting possible. But although the time might have gone when the two-dimensional journal article could suffice for complex papers, clinicians should nonetheless apply the same critical assessment that they would for any other clinical tool. If a result is too good to be true, it probably is.

